# Rate–Distortion–Perception Optimized Neural Speech Transmission System for High-Fidelity Semantic Communications †

**DOI:** 10.3390/s24103169

**Published:** 2024-05-16

**Authors:** Shengshi Yao, Zixuan Xiao, Kai Niu

**Affiliations:** 1 Key Laboratory of Universal Wireless Communications, Beijing University of Posts and Telecommunications, Beijing 100876, China; 2 Department of Broadband Communication, Peng Cheng Laboratory, Shenzhen 518066, China

**Keywords:** speech transmission, joint source–channel coding, semantic communications

## Abstract

We consider the problem of learned speech transmission. Existing methods have exploited joint source–channel coding (JSCC) to encode speech directly to transmitted symbols to improve the robustness over noisy channels. However, the fundamental limit of these methods is the failure of identification of content diversity across speech frames, leading to inefficient transmission. In this paper, we propose a novel neural speech transmission framework named *NST*. It can be optimized for superior rate–distortion–perception (RDP) performance toward the goal of high-fidelity semantic communication. Particularly, a learned entropy model assesses latent speech features to quantify the semantic content complexity, which facilitates the adaptive transmission rate allocation. NST enables a seamless integration of the source content with channel state information through variable-length joint source–channel coding, which maximizes the coding gain. Furthermore, we present a streaming variant of NST, which adopts causal coding based on sliding windows. Experimental results verify that NST outperforms existing speech transmission methods including separation-based and JSCC solutions in terms of RDP performance. Streaming NST achieves low-latency transmission with a slight quality degradation, which is tailored for real-time speech communication.

## 1. Introduction

The vast demand of streaming audio and video communication poses significant challenges to wireless communication systems, underscoring the need to elevate both the quality and efficiency of speech transmission. Current wireless communication systems suffers from the cliff effect where the signal reconstruction quality breaks down if the channel quality falls below the level anticipated by the channel code. Learning-based speech transmission methods [1,2,3] are emerging as promising solutions to improve the end-to-end transmission performance in the context of semantic communication [4,5,6,7]. They mostly leverage the idea of joint source–channel coding (JSCC) to produce transmitted symbols directly from raw speech signals with neural networks, which is featured with graceful degradation with respect to channel quality [1,2,8]. However, these approaches fail to identify the content diversity among signals, leading to inefficient transmission. Streaming inference is also a fundamental aspect in real-time communication (RTC) scenarios. Although transmission errors can be compensated by retransmission such as hybrid automatic repeat requests, these lead to a loss of efficiency and transmission delay.

To address the above-mentioned issues, we make the first attempt to design a high-fidelity neural speech transmission framework (*NST*) for better end-to-end transmission performance. Motivated by learned data compression techniques [9,10], NST establishes a learned entropy model on latent speech features and then realizes semantic-guided variable-length joint source–channel coding, thus achieving better coding gain. Specifically, a critical set of hyperprior variables is established upon the latent features, which estimate the entropy of speech features by variational modeling. Under the guidance, speech latent features are dynamically encoded to variable-length symbol sequences via a joint source–channel encoder. Based on our previous work [11], we further investigate the real-time speech transmission within latency-sensitive contexts, such as online conferencing and voice calls. In particular, we develop a streaming variant of NST tailored for low-latency transmission. All the operators of the model are strictly causal ones, which attend to the past speech signals only, to satisfy the real-time property. In addition, we design a sliding-window based inference mechanism in joint source–channel coding, which balances the performance of speech reconstruction and the overall delay.

We evaluate the performance by conducting simulations over wireless channels. The results demonstrate that the proposed NST model is source and channel-adaptive. In comparison to advanced speech coding combined with error correction coding, and the existing JSCC solution, the proposed NST achieves a superior rate–distortion–perception tradeoff. This translates to a high-fidelity speech reconstruction performance while incurring lower bandwidth costs. Notably, the streaming NST makes a slight compromise in speech quality to meet the low-latency requirement.

*Notational Conventions:* Throughout this paper, bold letters (e.g., x) denote vectors and the scalars, and lowercase ones denote scales. Bold uppercase letters (e.g., V) represent a collection. $\log\left(\cdot \right)$ is the logarithm to base 2. $p_{x}$ denotes a probability density function (pdf) with respect to the continuous-valued random variable *x*. U(a−m,a+m) denotes a uniform distribution centered on *a* with width 2m. R and C denote the real number set and the complex number set, respectively. $E\left[\cdot \right]$ denotes the statistical expectation operation.

## 2. Methodology

### 2.1. Architecture

**Figure 1:**
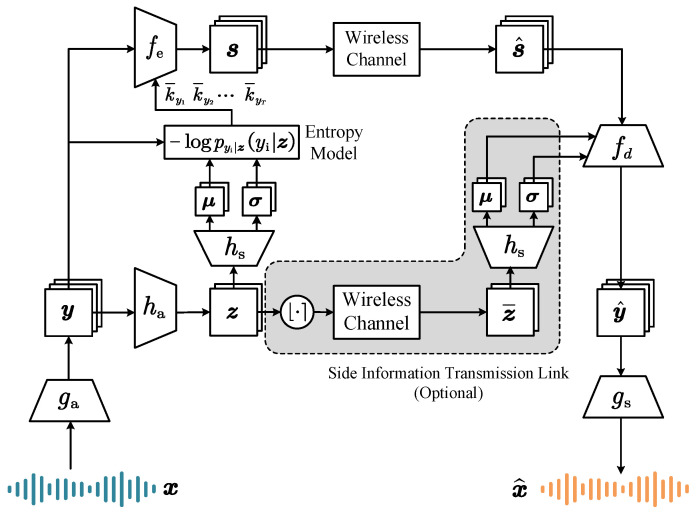
The architecture of the Neural Speech Transmission system (NST).

The NST system architecture is illustrated in Figure 1. Assuming a sequence of *T* speech frames $x=\left\{x_{1},x_{2},\cdots x_{T}\right\}$, the analysis transform module $g_{a}\left(\cdot ;\phi_{g}\right)$, which consists of convolutional neural networks (CNNs) with temporal downsampling, transforms them into a semantic latent feature sequence $y=\left\{y_{1},y_{2},\cdots y_{T}\right\}$. Then, the latent features y are fed into both a hyperprior encoder $h_{a}\left(\cdot ;\phi_{h}\right)$ and a variable-length JSCC encoder $f_{e}\left(\cdot ;\phi_{f}\right)$. On one hand, in order to conveniently quantify the amount of information for speech features, each element of y is variationally modeled by a simple Gaussian, whose parameters are encapsulated by the hyperprior variable z. The means and variances of the Gaussians are encoded by $h_{a}\left(\cdot ;\phi_{h}\right)$ and $h_{s}\left(\cdot ;\theta_{h}\right)$ to capture the dependencies of y. On the other hand, $f_{e}\left(\cdot ;\phi_{f}\right)$ encodes y into channel-input sequence $s=\left\{s_{1},s_{2},\cdots s_{T}\right\}$, where $s_{i}\in C^{k_{i}}$ is a $k_{i}$-dimensional complex vector to transmit $y_{i}$. We consider a wireless channel denoted by $W\left(\cdot ;\nu \right)$, where ν denotes the channel parameters. Thus, the receiver obtains the sequence $\hat{s}=W\left(s;\nu \right)$ with the transition probability $p_{\hat{s}|s}\left(\hat{s}|s\right)$. As illustrated in Figure 1, with a mirrored design, the JSCC decoder $f_{d}\left(\cdot ;\theta_{f}\right)$ reconstructs latent representation $\hat{y}$, and semantic synthesis transform $g_{s}\left(\cdot ;\theta_{g}\right)$ recovers speech waveform $\hat{x}$. Hence, the total link of *NST* is formulated by

(1) $$x\overset{g_{a}\left(\cdot ;\phi_{g}\right)}{\to }y\overset{f_{e}\left(\cdot ;\phi_{f}\right)}{\to }s\overset{W\left(\cdot ;\nu \right)}{\to }\hat{s}\overset{f_{d}\left(\cdot ;\theta_{f}\right)}{\to }\hat{y}\overset{g_{s}\left(\cdot ;\theta_{g}\right)}{\to }\hat{x},$$

with the latent prior $y\overset{h_{a}\left(\cdot ;\phi_{h}\right)}{\to }z\overset{h_{s}\left(\cdot \theta_{h}\right)}{\to }\left\{\mu ,\sigma \right\}$ and $\left(\theta ,\phi \right)=\left(\phi_{g},\phi_{h},\phi_{f},\theta_{g},\theta_{h},\theta_{f}\right)$ encapsulating the learnable parameters of each function above. Moreover, the hyperprior z can be viewed as side information, which is optionally sent via a digital link to the receiver to refine the latent feature y.

### 2.2. Dynamic Variable-Length Joint Source–Channel Coding

As defined previously, each $y_{i}$ is variationally modeled as a Gaussian with mean $\mu_{i}$ and variance $\sigma_{i}^{2}$, whose density function is factorized as

(2) $$p\left(y|z;\theta_{h},\psi_{h}\right)=\prod_{i}\underset{p_{y_{i}|z}}{\underset{︸}{\left(N\left(\mu_{i},\sigma_{i}^{2}\right)*U\left(-\frac{1}{2},+\frac{1}{2}\right)\right)}}\left(y_{i}\right),$$

with $\left(\mu ,\sigma \right)=h_{s}\left(z\right)$, where ∗ is a convolutional operation. Dithered quantization is adopted [12], such that we can derive a non-negative entropy estimation of $-\log p_{y|z}\left(y|z\right)$ by directly using the proxy ${\tilde{y}}_{i}=y_{i}+o,o\in U\left(-\frac{1}{2},+\frac{1}{2}\right)$. The estimated entropy is directly linked to the channel bandwidth cost in the JSCC encoder for transmission. Intuitively, if $y_{i}$ is tagged with high entropy, it will be allocated with more bandwidth and vice versa.

In practice, the total bandwidth cost $K_{y}$ for transmitting y is formulated by

(3) $$K_{y}=\sum_{i=1}^{T}{\bar{k}}_{y_{i}}=\sum_{i=1}^{T}Q\left(k_{y_{i}}\right)=\sum_{i=1}^{T}Q\left(-\eta_{y}\log p_{y_{i}|z}\left(y_{i}|z\right)\right),$$

where $\eta_{y}$ controls the scaling between the estimated entropy and the number of transmitted symbols, and *Q* denotes a $2^{n}$-level scalar quantization with the quantized value set as $V=\{v_{1},v_{2},\cdots ,v_{2^{n}}\}$. Hence, *n* bits are transmitted as side information to inform the receiver in which ${\bar{k}}_{y_{i}}\in V$ is selected for transmitting $y_{i}$.

**Figure 2:**
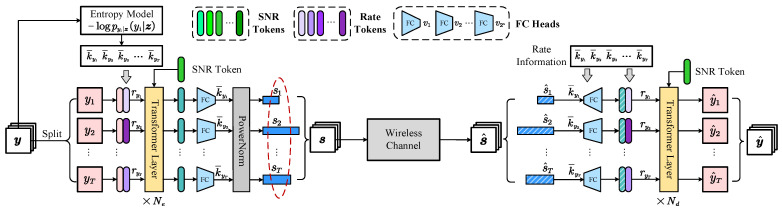
The pipeline of the variable-length joint source-channel coding (JSCC) via $f_{e}$ and $f_{d}$. FC denotes fully connected layers.

**Figure 3:**
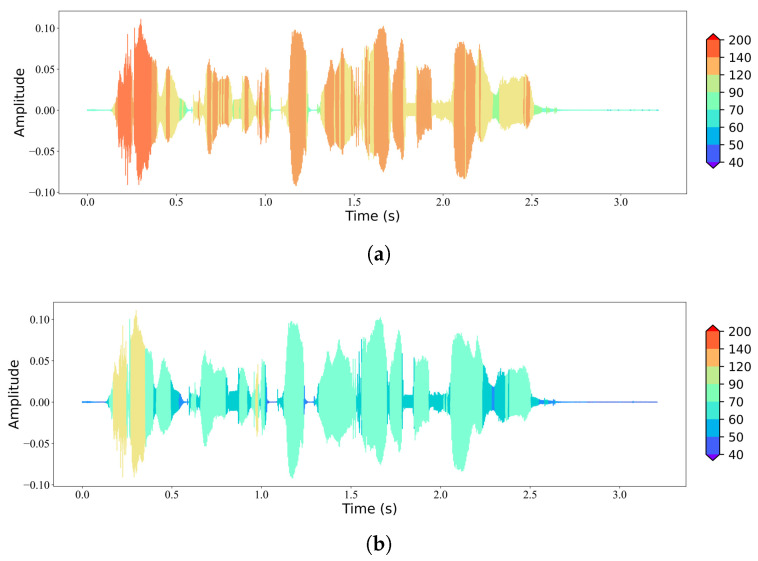
Visualization of rate allocation along temporal domain. (**a**) Bandwidth *K* = 10 kHz with $\eta_{y}$ = 0.16. (**b**) Bandwidth *K* = 4 kHz with $\eta_{y}$ = 0.105.

We adopt a pair consisting of a Transformer-like [13] JSCC encoder and decoder as $f_{e}$ and $f_{d}$, as plotted in Figure 2. Guided by the entropy model $-\log p_{y|z}\left(y|z\right)$, a set of learnable rate token embeddings with the same dimension with $y_{i}$ are developed, each of which corresponds to a value in V. To adapt to various channel environments, we assume a channel state information feedback to inform the sender of the instant signal-to-noise ratio (SNR). Similarly, a set of learnable SNR tokens are developed. *T* frames of speech features are gathered and fused with respective rate tokens and an SNR token, and they are finally fed into the Transformer block with $N_{e}$ Transformer layers. A bunch of fully connected (FC) layers with output dimensions of $v_{q},q=1,2,\cdots ,2^{n}$ are employed to map the embeddings into $s_{i}$ with given dimensions. A toy visualization of the rate allocation result is displayed in Figure 3. It can be observed that more bandwidth is allocated to frames with prominent contents and less is allocated to ones in silence. The overall bandwidth is adjusted by tuning the hyperparameter $\eta_{y}$.

### 2.3. Streaming NST for Real-Time Communication

In this subsection, we propose a streaming NST model variant to facilitate real-time speech communication.

Firstly, all the convolutional operators in $g_{a}$ and $g_{s}$ are substituted by causal ones. The transposed convolution operations in $g_{s}$ only pad on the past steps to meet the causal property. Secondly, the joint source–channel coding of speech latent features is modified from that in Figure 3 to reduce the latency. Traditionally, Transformer uses multi-head attention that jointly learns diverse relationships between queries and keys, which are the speech features y in this paper, from different representation subspaces with *j*-th head computing

(4) $$Q_{j}=W_{j}^{Q}y,K_{j}=W_{j}^{K}y,V_{j}=W_{j}^{V}y.$$

**Figure 4:**
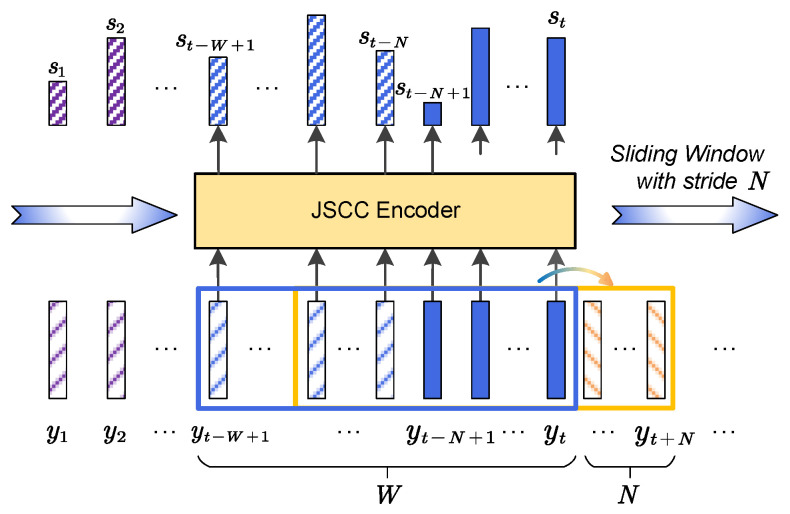
Streaming joint source–channel encoding for real-time inference. It encodes latent features of *N* frames into transmitted symbols in each inference, e.g., $y_{t-N+1},\cdots ,y_{t}$ for the blue window in the figure and then the ones in orange in the next inference.

To meet the real-time requirement, a causal masked attention method is proposed together with a sliding-window inference mechanism. As shown in Figure 4, the JSCC encoder $f_{e}$ has a limited contextual window with *W* frames, which hops along the temporal domain with a stride of *N* frames. In particular, we follow Transformer-XL [14] and create a segment-level recurrence of output by intermediate layers. In this paper, we define the attention span of each layer as 2N frames, which ends with the last frame of the current *N* target frames. The self-attention is computed after a causal mask M is applied, whose elements satisfy

(5) $$M_{t,\tau }=\left\{\begin{array}{cc} 1, & t-2N<\tau \leq t\\ -\infty , & \mathrm{others} \end{array}\right..$$

Then, the output of the *j*-th head self-attention $a_{j}$ is formulated by

(6) $$a_{j}=\mathrm{Softmax}\left(\frac{Q_{j}K_{j}^{T}}{\sqrt{d_{h}}}⊙M\right)V_{j},$$

where $d_{h}$ is the dimension of each head. Thus, the length of contextual window *W* grows linearly with respect to the number of Transformer layers as well as the window stride, which can be written as $W=N(N_{e}+1)$ frames.

### 2.4. Optimization Goal

The analysis transform together with the joint source–channel encoder creates a parametric density $q_{\hat{s},\tilde{z}|x}$ to approximate the true posterior distribution $p_{\hat{s},\tilde{z}|x}$. The optimization goal is to minimize the Kullback–Leibler (KL) divergence between the above two components. After reformulation, it minimizes its upper bound, i.e.,

(7) $$\min\underset{x∼p_{x}}{E}\underset{\hat{s},\tilde{z}∼q_{\hat{s},\tilde{z}}}{E}D_{\mathrm{KL}}\left[q_{\hat{s},\tilde{z}|x}∥p_{\hat{s},\tilde{z}|x}\right]\leq \min\underset{x∼p_{x}}{E}\underset{\hat{s},\tilde{z}∼q_{\hat{s},\tilde{z}}}{E}\left[\underset{\mathrm{side}\;\mathrm{info}.\;\mathrm{coding}\;\mathrm{rate}}{\underset{︸}{-\log p_{\tilde{z}}\left(\tilde{z}\right)}}\underset{\mathrm{bandwidth}}{\underset{︸}{-\log p_{\hat{s}|\tilde{z}}\left(\hat{s}|\tilde{z}\right)}}\underset{\mathrm{distortion}}{\underset{︸}{-E_{y∼p_{y|\hat{s},\tilde{z}}}\log p_{x|y}\left(x|y\right)}}\right]+\mathrm{const}.$$

The first term of (7) represents the cost of encoding the side information assuming $p_{\tilde{z}}$ as the entropy model, where ${\tilde{z}}_{i}=z_{i}+o$ is the proxy quantization of $z_{i}$. Since there is no prior information about z, $p_{\tilde{z}}\left(\tilde{z}\right)$ is modeled as a non-parametric fully factorized density [9]$p_{\tilde{z}}\left(\tilde{z}\right)=\prod_{i}\left(p_{z_{i}|\psi^{\left(i\right)}}\left(z_{i}|\psi^{\left(i\right)}\right)*U\left(-\frac{1}{2},\frac{1}{2}\right)\right)\left(z_{i}\right)$. The second term represents the bandwidth cost of encoding $\hat{s}$. In practice, the intermediate variable y is utilized by $p_{\hat{s}|\tilde{z}}=W\left(p_{s|\tilde{z}}|h\right)=W\left(f_{e}\left(p_{y|\tilde{z}}\right)|h\right)$. The third term denotes the weighted distortion of the reconstructed speech waveform. d(·,·) indicates the objective signal distortion. To enrich the distortion term in alignment with human perceptual quality, a differentiable F(·) is employed as a perceptual feature extractor, and the distance between perceptual features $d_{p}\left(\cdot ,\cdot \right)$ is minimized to improve the listening quality.

In summary, the RDP function is formulated as

(8) $$L_{\mathrm{RDP}}\left(\theta ,\phi ,\psi \right)=E_{x∼p_{x}}\left[-\eta_{y}\log p_{y|z}\left(y|z\right)-\eta_{z}\log p_{\tilde{z}}\left(\tilde{z}\right)+\lambda_{D}d\left(x,\hat{x}\right)+\lambda_{P}d_{p}\left(F_{x},F_{\hat{x}}\right)\right],$$

where the Lagrange multipliers $\lambda_{D},\lambda_{P}$ control the tradeoff among the total transmission rate, the distortion and the perceptual quality. The scaling factor $\eta_{y}$ is adjusted for RDP tradeoff, while $\eta_{z}$ is determined according to the channel capacity of the optional tranmission link.

## 3. Results

In this section, we provide numerical results in terms of objective quality metrics and subjective scores to evaluate the quality of speech transmission.

### 3.1. Experimental Setup

The mono speech signals are sampled at 16 kHz from the TIMIT dataset [15]. Compared to our conference paper [11], to adapt to RTC scenarios, a shorter frame length is considered in this paper. Each speech frame has L=128 samples with an overlap of eight samples. The analysis transform module $g_{a}$ and synthesis transform module $g_{s}$ consist of stacks of 1D convolutional layers with a residual connection. The number of channels of the convolutional kernel of the output/input layer for $g_{a}$/$g_{s}$ is configured with $C_{g}=4$, while the one for $h_{a}$/$h_{s}$ is set as $C_{h}=2$. In the variable-length JSCC coder $f_{e}$ and $f_{d}$, we use $N_{e}=3$ Transformer layers with eight-head self-attention. The quantized channel bandwidth cost value set is defined as $V=\left\{10,40,90,120,200,250,300,400\right\}$. Each speech frame $x_{i}\in R^{1\times L}$ is transformed into latent feature $y_{i}\in R^{C_{g}\times \frac{L}{4}}$ with a downsampling factor of four. It is then flattened into an embedding vector with a dimension of $\frac{C_{g}L}{4}=128$, which is identical to the dimension of the Transformer in JSCC coders.

In (8), the object signal distortion *d* is evaluated by the mean square error in the time domain. In terms of perceptual optimization, we minimize the difference of Mel-frequency cepstral coefficients (MFCCs) [16], which is a hand-crafted speech perceptual feature. Specifically, a mean square loss function $d_{p}$ for MFCCs is employed, where *F* denotes the function of the MFCC extractor.

We compare our NST model with traditional separation-based transmission schemes. Specifically, we employ the widely used speech codec AMR-WB [17] and Opus [18] for source coding and convolutional codes, 5G LDPC [19] for channel coding, and follow the principle of adaptive modulation coding (AMC) [20]. Moreover, we also compare our NST model with another JSCC model DeepSC-S [1] for speech transmission, which is a non-streaming model with CNN modules. We modify its model to support low bandwidth transmission with 12 kHz and 32 kHz, separately.

### 3.2. Evaluation Metrics

In terms of objective metrics for perceptual quality, we report the perceptual evaluation of speech quality (PESQ) [21] scores, which range from 1.0 to 4.5. Furthermore, we implement a Multiple Stimuli with Hidden Reference and Anchor (MUSHRA) subjective test [22] for human preference evaluation. As a widely used approach in the subject quality assessment method, the MUSHRA test allows users to compare multiple variants of reconstructed audio and provides the relative score between 0 and 100. We randomly select 10 speech segments from the test set.

### 3.3. Results Analysis

**Figure 5:**
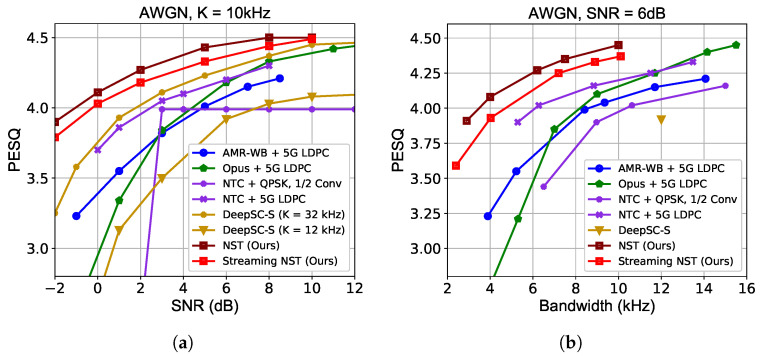
Perceptual evaluation of speech quality (PESQ) performance over additive white Gaussian noise (AWGN) channel. (**a**) PESQ scores versus signal-to-noise ratio (SNR). The bandwidth of all methods *K* is 10 kHz, except those of DeepSC-S are 12 kHz and 32 kHz (yellow lines). (**b**) PESQ scores versus channel bandwidth cost when SNR = 6 dB.

Figure 5 reports the PESQ performance over additive white Gaussian noise (AWGN) channels. In Figure 5a, with a fixed channel bandwidth cost of K= 10 kHz, we find that the proposed NST brings a performance gain for all SNRs by incorporating source and channel information into JSCC, especially in a low SNR region. In addition to traditional speech source coding methods, we also compare with a nonlinear neural speech compressor which employs the similar entropy model as NST to entropy encode the latent speech features. This scheme is marked in the figure as “NTC + QPSK, 1/2 Conv” when using convolutional codes with a rate of 1/2 and QPSK modulation as “NTC + 5G LDPC” when using LDPC codes. NST demonstrates graceful performance degradation with the decrease of SNR, while the performance of other separation-based methods probably breaks down (cliff effect) when using a single-channel coding rate and modulation level, e.g., “NTC + QPSK, 1/2 Conv”. For the 5G LDPC, we plot the envelope of several curves, corresponding to different coding rates and modulation levels. Compared with another JSCC method DeepSC-S, our model achieves better perceptual quality by introducing an explicit perceptual loss function with much less bandwidth cost. In addition, we notice a slight quality drop under the streaming inference setting, but it remains better than other methods. NST adapts well to various channel conditions by means of SNR token fusion in $f_{e}$ and $f_{d}$ using a single model.

Figure 5b compares the rate–distortion–perception performance using different methods for the 6 dB AWGN channel. Since the NST model learns an adaptive rate allocation mechanism, we traverse the $\eta_{y}$ from 0.1 to 0.3 and finetune the model with a fixed $\lambda_{D}$ and $\lambda_{P}$. It can be observed that a remarkable bandwidth saving can be accomplished for NST by integrating source semantic information as well as channel information.

**Figure 6:**
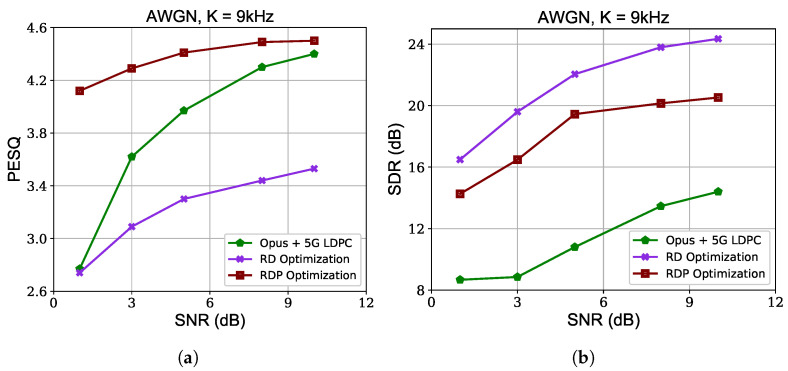
Distortion–perception tradeoff using different optimization objectives with 9 kHz channel bandwidth cost over AWGN channel. (**a**) PESQ for assessing perceptual quality. (**b**) Signal–to–distortion ratio (SDR) for assessing signal distortion.

To delineate the distortion–perception tradeoff, we conduct an ablation study examining the impact of perceptual optimization. We evaluate the signal–to–distortion ratio (SDR) performances to assess the traditional signal distortion. The results in Figure 6 demonstrate that the proposed NST using an RDP optimization objective function (8) outperforms its counterpart solely optimized toward reduced signal distortion in terms of perceptual quality. NST with rate–distortion (RD) optimization (omitting perceptual loss in (8)) exhibits inferior perceptual quality despite there being less objective signal distortion. Performance using the traditional speech coding method is also included in the figure, which also underscores the significance of perceptual optimization in addition to minimizing the objective signal distortion.

**Figure 7:**
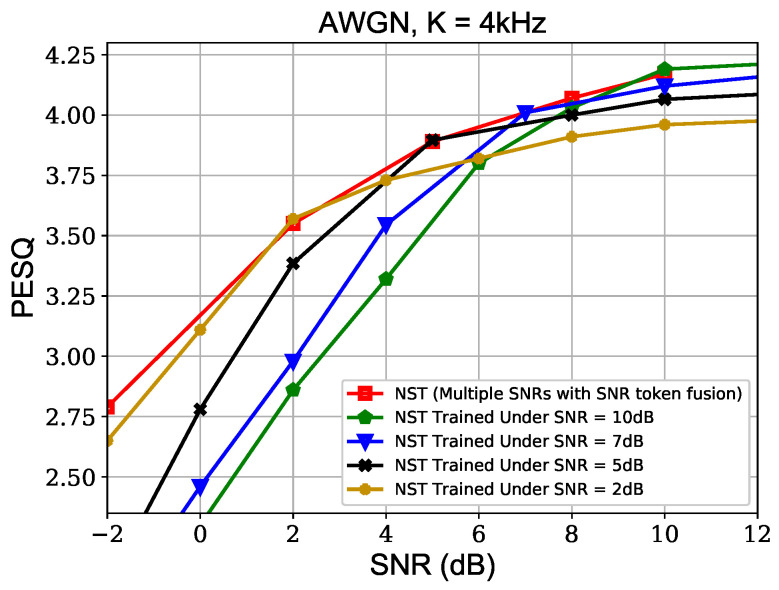
Effect of SNR token fusion in joint source–channel coding.

Figure 7 displays the effect of SNR fusion in our SNR-adaptive joint source–channel coding. It can be observed that the PESQ-SNR curve of the proposed NST trained under multiple SNRs with SNR token fusion closely approximates the envelope of the curves obtained from models trained using single SNR values.

**Figure 8:**
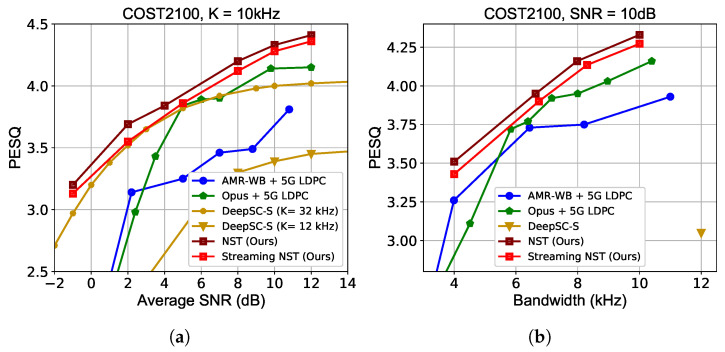
PESQ performance over COST2100 fading channel. (**a**) PESQ scores versus average SNR. (**b**) PESQ scores versus channel bandwidth cost.

We additionally carry out experiments on the widely used COST2100 fading channel [23] to verify the robustness of the NST model. Figure 8 shows the results. With a feedback of average SNR and the SNR token fusion, our model adapts to the channel states well, while performances of DeepSC-S are evaluated on models trained at multiple SNRs. With lower bandwidth in Figure 8, NST also shows better transmission efficiency compared to traditional methods.

**Figure 9:**
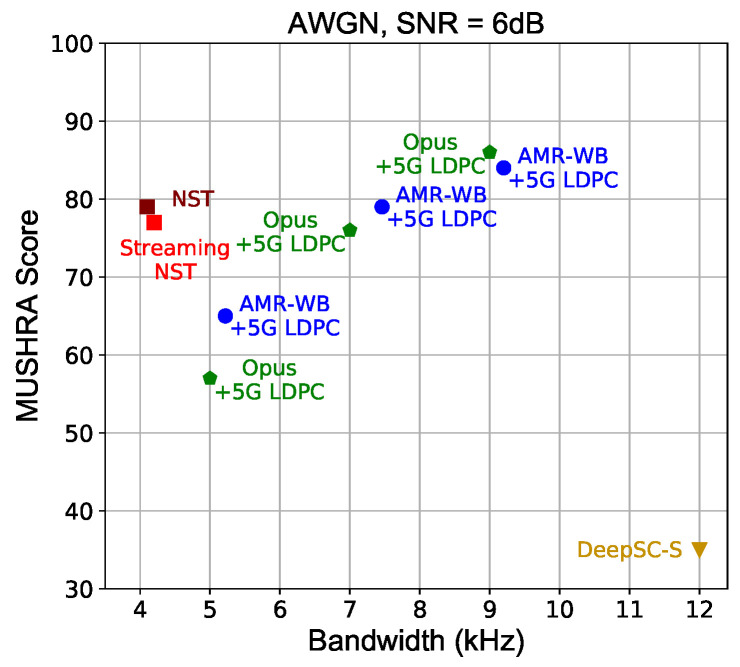
MUSHRA scores evluated under 6 dB AWGN channel. Audio samples are available at https://ximoo123.github.io/NSTSpeech (accessed on 1 March 2024).

The subjective user rating results in Figure 9 verify that the proposed NST recovers perceptually satisfying speech over 6 dB AWGN channels, even consuming much less bandwidth than separation-based speech coding methods. Compared to DeepSC-S, which is only optimized for lower distortion, the RDP-optimized NST achieves semantics-guided dynamic rate allocation, thus much improving the end-to-end system gain. The perceptual quality of streaming NST exhibits no substantial degradation compared to the non-streaming one, which is of practical value in RTC scenarios.

### 3.4. Discussion on the Quality–Latency Tradeoff

In terms of streaming NST, we investigate the tradeoff between the perceptual quality and the transmission latency. As is defined previously, each frame of speech feature $y_{i}$ accounts for 8 milliseconds (ms) of a 16 kHz signal.

Table 1 shows the tradeoff between speech quality and transmission delay, which consists of encoding and decoding time (runtime) and the latency. In the context of sliding-window-based inference, a longer stride will increase the latency as it needs to wait for the arrival of future frames to collect all features belonging to the same window.

**Figure 10:**
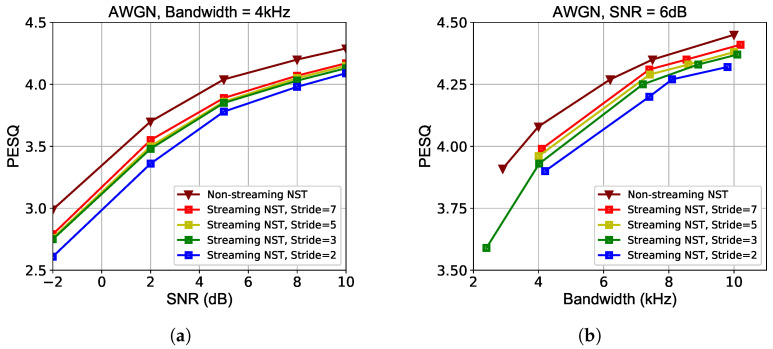
PESQ performances using different strides *N* over AWGN channels. (**a**) PESQ scores versus SNR. (**b**) PESQ scores versus bandwidth.

We also compare the PESQ performances versus stride frames across different SNRs and bandwidth cost. The results in Figure 10 verify that a longer window stride as well as the length of contextual windows in JSCC consistently presents a better coding gain across different transmission conditions at the cost of longer delay according to Table 1. Except this subsection, performances of streaming NST are reported with a stride of N=3 and a total delay of less than 100 ms. It satisfies the real-time property and ensures a high-quality speech restoration simultaneously. The runtime is evaluated on an Intel(R) Core i9-12900K CPU (Intel Corporation, Santa Clara, CA, USA). Table 2 presents the model complexity comparison of both computational (measured by giga floating point operations per second, i.e., GFLOPs) and space complexity. Due to the employment of a tiny Transformer in the joint source–channel encoder, our model is comparably lightweight and computational efficient. Extra measures for accelerating inference may be taken to facilitate lightweight deployment in resource-limited devices.

## 4. Conclusions

In this paper, we present the NST, which is a novel neural speech transmission framework. The model features dynamic rate allocation for variable-length JSCC, which is guided by the variational modeling of speech latent features. It presents good adaptability to varying channel conditions by channel information fusing in JSCC. A streaming variant of NST is also designed for RTC. Simulation results verify that the proposed method consumes much less bandwidth cost than classical methods when achieving similar perceptual performances. It highlights NST’s potential in high-efficiency and high-fidelity speech transmission in the realm of semantic communication.

## Figures and Tables

**Table 1:** Quality–delay tradeoff for the streaming NST model tested with SNR = 10 dB over the AWGN channel.

Stride	PESQ	Total Delay	Runtime	Maximum Latency
2	4.09	67.1 ms	51.1 ms	16 ms
3	4.13	83.2 ms	59.2 ms	24 ms
5	4.15	112.7 ms	72.7 ms	40 ms
7	4.17	140.1 ms	84.1 ms	56 ms

**Table 2:** Model complexity comparison.

Model	GFLOPs	#Params (Unit: Million)
[3]	>31	>106
DeepSC-S [1]	7.60	0.24
NST (Ours)	9.87	2.49

## Data Availability

No new data were created or analyzed in this study. Data sharing is not applicable to this article.
